# Family medicine residency training and burnout: a qualitative study

**Published:** 2014-12-17

**Authors:** Kimberly Rutherford, Joanna Oda

**Affiliations:** 1 Department of Family Practice, University of British Columbia, Vancouver, British Columbia, Canada

## Abstract

**Background:**

Almost three-quarters of family practice residents in British Columbia (BC) meet criteria for burnout. We sought to understand how burnout is perceived and experienced by family medicine residents, and to identify both contributory and protective factors for resident burnout.

**Method:**

Two semi-structured focus groups were conducted with ten family practice residents from five distinct University of British Columbia training sites. Participants completed the Maslach Burnout Inventory (MBI). The data were analyzed using a thematic analysis approach.

**Results:**

Seventy percent of the focus group participants met criteria for burnout using the MBI. The experience of burnout was described as physical and emotional exhaustion, loss of motivation, isolation from loved ones, and disillusionment with the medical profession. Contributory factors included high workload, burned-out colleagues, perceived undervaluing of family medicine, lack of autonomy, and inability to achieve work-life balance. Protective factors included strong role models in medicine, feeling that one’s work is valued and rotations in family medicine.

**Conclusions:**

The high level of burnout in family medicine residents in BC is a multifactorial and complex phenomenon. Training programs and faculty should be aware of burnout risk factors and strive to implement changes to reduce burnout, including allowing residents increased control over scheduling, access to counseling services and training for resident mentors.

## Introduction

Burnout is a psychological syndrome characterized by emotional exhaustion, depersonalization or cynicism and a reduced sense of personal accomplishment that develops in response to prolonged occupational stress and depletion of personal coping resources.^1^ The Maslach Burnout Inventory (MBI) is a questionnaire that measures burnout along its three dimensions: emotional exhaustion, depersonalization and personal accomplishment, and has been validated for use among physicians and residents.^2^ Using the MBI, studies have estimated that between 18% and 76% of medical residents are burned out.^3^–^5^ The prevalence of burnout in residents appears to be higher than that of the general population,^6^ and high levels have been reported in many countries, across a wide range of medical and surgical specialties, and in all years of training.^3^–^5^,^7^–^13^

Burnout has serious consequences for both medical residents and the patients under their care. Residents meeting criteria for burnout in a Dutch study reported poorer physical health than those who were not burnt out.^14^ Burnout was also associated with depression in this study; of the residents meeting criteria for burnout, 25% were also depressed, while 96% of depressed residents also met criteria for burnout. Further, the prevalence of suicidal thoughts in burned out residents has been shown to be more than double that seen in non burned out residents.^9^ The burnout experienced by residents also affects patient care. Shanafelt *et al.* showed that residents meeting criteria for burnout were two to three times more likely than their non burned out colleagues to report suboptimal patient care practices at least monthly.^5^ Similarly, West *et al.* also found burnout to be independently associated with higher rates of self-perceived major medical errors.^15^

Despite a growing body of literature on burnout, studies report conflicting results regarding potential contributory and protective factors.^3^,^16^ A 2004 review by Thomas was unable to identify any demographic or personality features that could reliably predict residents at-risk for burnout.^8^ Some studies report financial stress contributing to burnout,^3^,^17^ and financial security as protective;^18^ others, however, find no such associations.^19^ A 2009 review by Ishak *et al.* described time demands, lack of control over time management and inherently difficult job situations as commonly cited contributors to burnout.^16^ Lack of support systems^19^ and dissatisfaction with time for leisure and exercise^18^ have also been identified as contributing to burnout. The impact of family/personal relationships on burnout is more complex; one study showed lack of time with family and friends associated with burnout,^20^ while the review by Prins *et al*. reported higher levels of burnout in both unmarried residents and those experiencing work-home conflict or family-related stress.^3^

Notably, the majority of studies on burnout employ quantitative methods and identify risk and protective factors by correlating survey responses or demographic data with MBI scores. For example, one study reported 27 factors that were significantly associated with at least one domain of burnout; however the cross-sectional survey method used prohibits inferring causation.^18^ Qualitative methods could shed light on these factors and other conflicting results. Yet the authors could identify only one qualitative study of resident physician burnout: Satterfield *et al.* retrospectively analyzed the progress notes of six internal medicine resident support groups to identify common themes, stressors, emotions and coping strategies. Dominant themes reported included the importance of peer relationships, feelings of anxiety and guilt, uncertainty regarding the resident role and responsibilities and the development of professional confidence.^21^

Studies investigating risk factors for burnout in family practice training are scarce compared to other specialties.^8^,^16^ A 1988 study of family practice residents in the United States found no correlation between burnout and any demographic characteristics;^19^ “time demands” was the factor most often cited as contributing to burnout.^22^ A 2013 study of French general practitioners in training reported the following factors associated with burnout: time spent working, rotations in internal medicine, lack of recognition from senior physicians and dissatisfaction with time for family, friends and leisure.^20^

A survey of University of British Columbia (UBC) family medicine residents in 2011 found that 74% of residents met criteria for burnout, defined as either a high emotional exhaustion or depersonalization score on the MBI.^23^ In order to design interventions to address these high rates of burnout in BC and elsewhere, a thorough understanding of both protective and contributory factors is essential. The purpose of this study is to qualitatively explore the experiences of burnout as they relate to family medicine training, to identify factors that contribute to and protect against burnout, and to elicit suggestions for decreasing burnout during residency training. This study adds qualitative results to the mostly quantitative data landscape on resident burnout and contributes to the limited literature on burnout in family practice training.

## Methods

### Study design

A focus group format was selected for this study, as this method is useful for exploring attitudes and needs of health care workers.^24^ With few qualitative studies on the topic, an inductive thematic analysis approach was selected for this exploratory study. A preconceived theoretical framework was not used; the researchers allowed themes to emerge as the data were analyzed. This study was approved by the UBC Research Ethics Board.

### Participants

All 237 family medicine residents in British Columbia were invited to participate in this study via email. After reviewing the study information and focus group times, ten were able to participate. Inclusion criteria involved being a current resident in the UBC Department of Family Practice. Self-identifying as experiencing burnout was not an inclusion criteria. Participants signed a written consent form prior to participating in a focus group. Residents outside of Vancouver were offered the opportunity to participate by teleconference.

### Data collection

Two semi-structured focus groups, facilitated by the authors, were held at St. Paul’s Hospital in Vancouver in March, 2012 and were 70 and 85 minutes in length. Nine residents participated in person and one by video teleconference. Each participant completed the MBI prior to the focus group. The research team obtained the appropriate license to administer the MBI. The MBI is composed of 16 statements of job-related feelings that participants rate on a numerical frequency scale between ‘0’/‘never’ and ‘6’/‘every day.’ Each question contributes to one of the three burnout domains and participant ratings are tallied to give a numerical score for each domain which is categorized as ‘high,’ ‘moderate,’ or ‘low’ according to a scoring key. A flexible interview guide was used to facilitate the discussion. Prompts were related to the four main research questions: 1) residents’ experiences of burnout, 2) perceived risk factors, 3) perceived protective factors, and 4) suggestions for preventing burnout. Focus groups were audio recorded and transcribed verbatim with identifying features removed.

### Data analysis

Data were analyzed for themes using a thematic analysis approach. Transcripts were independently read and coded by the two authors. Discussion and comparison of coding led to the identification of themes and the development of a preliminary thematic framework. This was reviewed by the research team and revised through debate and discussion with ongoing review of the transcripts in the context of larger emerging themes. A final code book was agreed upon that included clear definitions of themes and sub-themes. Transcripts were independently coded by the two authors who met to review and discuss any differences in coding. A final, unified coded dataset was negotiated and data collected under each theme summarized into an analytic memo.

## Results

**Figure 1:**
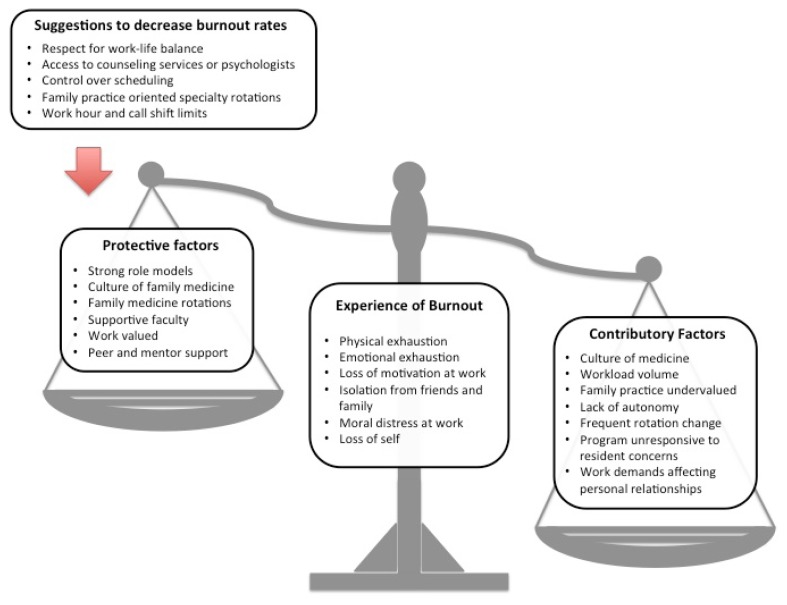
Schematic representation of the themes identified through data analysis

The ten study participants represented five UBC training sites; demographic data are presented in Table 1. Using the MBI, 70% of the residents participating in the focus groups met criteria for burnout. This is similar to the overall burnout rate of 74% reported in a 2011 program-wide survey.^23^ Mean scores for residents meeting the criteria for burnout in 2012 and the corresponding categories are shown in Table 2. MBI results for both groups are compared in Table 3. Themes identified during data analysis are presented according to the four main research questions. Figure 1 shows a schematic representation of the study findings.

### The experience of burnout

While only 70% of participants met criteria for burnout by MBI score, all participants identified feeling ‘burned out’ at some point during residency training. Participants described significant physical and emotional exhaustion during residency that affected their ability to learn and negatively impacted their interactions with patients.

You’re tired, you’ve got a list of other things to do and I think that’s why you lose sight sometimes of the patient - and that’s when it’s bad. (P6)

Many residents described feelings of guilt, cynicism and inadequacy at work.

I think for me, the part of it that was challenging was the feeling of, like, futility … that I wasn’t really contributing anything. (P5)

I would dread going in the morning… because I was like “why? what’s the point? I’m not learning, I’m not doing anything for anybody that’s useful” and I’d just get overwhelmed with those kinds of emotions and thoughts. (P6)

A number of residents also described distress at being expected to act or make decisions at work that they felt were contrary to their personal beliefs or values.

I just [have a] really overwhelming feeling that the things that brought me to medicine and things that I believed in and loved and, like, really valued in life were not being valued by the medical cultures and, yeah, it made me really cynical. (P10)

For many residents, feeling burned out related to isolation from friends and family. One aspect was physical isolation due to long work hours. Some participants described missing important events and celebrations, while one resident moved across the country for residency and felt she neither had time to make new friends, nor to keep in touch with old friends. Isolation was also described as a growing emotional distance between participants and their loved ones, who were unable to relate to their residency experiences.

[My partner] couldn’t understand what I was going through and sometimes I would come home and be like ‘I want to tell you about this really hard thing but you won’t understand and it’s going to upset you’… It was the first time that I felt like really like our paths had taken separate ways. (P5)

Even when I'm really exhausted - that's probably when you really need support the most - I don't really feel like I wanna reach out because I really have nothing to discuss. So … I find it quite difficult to keep up with friends. (P8)

Finally, many participants described a sense of loss of self that occurred during residency training. This included not having time for activities they once enjoyed and feeling that they had been changed by the process and experience of residency.

I noticed at that point like I've really changed ... I realize I've lost a bit more of the charitable outlook that I may have had earlier and I feel like I've become a bit more cynical, just kind of being the peon… I sometimes feel like I'm a cog in a wheel. I have no confidence in my skills a lot of the time so I think, like, a lot of these things are signs of feeling burned out. (P9)

### Risk factors for burnout

Participants described the medical culture they worked in as contributing to burnout in a variety of ways. One participant noted that burnout seemed to be ubiquitous at work. Some residents were disillusioned by the behavior and comments of their colleagues.

Some of the things that people said, it was so upsetting to me. Like, the way that people talked about their patients and, like, just the negativity. (P5)

Participants also described the undervaluing of family physicians and the practice of family medicine during their training.

As if [family medicine] is some kind of lesser field or something. Like, ‘Oh poor you, you have to be a family doctor and look after all these people after we’ve, kind of, discharged them’. (P1)

The lack of a space to debrief after a medical error was identified as particularly problematic.

I don't think that there's really a venue for physicians in general to get support around making mistakes and learning from them … I think that if we just, kind of, bury those mistakes in our psyche and build up guilt, that [it] will contribute to further burnout. (P7)

Rotations with high workload were identified as more likely to cause burnout.

I don’t usually find that, um, it’s the time itself at work that bothers me, it’s the workload when I’m at work … that intensity and that responsibility was bigger than the time I spent there. (P4)

Several residents described situations where their team was unable to accommodate their needs related to pregnancy, children, or illness, thus exacerbating the impact of heavy workloads. One resident expressed distress that her family could not rely on her as a result of her unpredictable work hours. Many residents felt frustrated by the lack of control over their schedules, and the impact of this on their personal lives and relationships contributed to burnout.

I paid for this soccer team and I love going to it, and it’s, like, my break for the week, but I don’t have access to that this week and next week and the week after that… because I just can’t get the time off and I have no control over that. Which tends to make me feel less supported. (P4)

Rotations where residents felt a mismatch between their level of supervision, knowledge and responsibility were also identified as contributing to burnout. These rotations added to feelings of inadequacy, guilt and anxiety.

The first couple rotations where I had full responsibility and very little guidance… [I] felt very burned out because I didn’t know [enough], didn’t feel comfortable with the patient care and [felt] that I wasn’t handling it well. (P6)

Frequent rotation changes caused residents to feel incompetent and hindered them from forming relationships with patients, attending physicians and other members of the healthcare team, thus contributing to burnout.

I think this comfort and support takes time to, to have. You can't get it on the first day of a rotation, uh, but you know, by the third week you know the staff, you know the residents, you're kind of getting settled [and] it's time for you to leave again. (P9)

The discord between frequent rotation changes and the goal of continuity of care in family medicine was underscored. Residents reported frustration during rotations where discrepancies arose between their learning goals and opportunities. One resident gave an example from his ICU rotation.

I would forget about my objectives of learning. Okay ‘I came here to do few central lines and to learn some other monitoring stuff.’ Uh, but it feel[s] divert[ed] into what the others - my senior, or my attending - is expecting because at the end of the day, I have to pass this rotation. (P2)

Finally, residency programs’ unresponsiveness to resident concerns and feedback was also identified as contributing to burnout. One resident described such unresponsiveness as:

A huge, huge, factor towards burnout, because you just feel like no one's listening, and no one's really looking out for us, and no one's really caring about our experience in the program. (P10)

Participants identified two personal factors contributing to burnout: an internal drive to excel and the difficulty balancing personal and work life. They described the challenge of building and maintaining personal relationships due to time constraints and emotional exhaustion.

All my relationships feel a little emptier this year, like they're superficial because I don't feel like I'm engaged with anyone or anything because I'm, like, in managerial resident mode. (P9)

### Factors protective against burnout

Residents identified having a supportive medical community with positive role models and mentors as protective against burnout.

Something I've found helpful in terms of trying to, um, keep cynicism away is having… role models around. For example, my family preceptor, she has a very good work-life balance. (P8)

Residents also felt protected against burnout when they knew their work was valued by their patients, preceptors and coworkers. The culture and nature of family medicine was also described as protective against burnout. Participants felt the discipline attracted physicians who valued and strived to achieve work-life balance.

The flexibility of family medicine is really amazing to prevent that burnout and the cynicism. You can do all the things that you are passionate about, that you enjoy and believe in. (P10)

Rotations in family medicine (both longitudinal and block components) were identified as highly protective, and a time when residents felt an increased sense of personal accomplishment.

By the end you’re starting to feel good. You know your patients, your patients know you, you know where everything is, you know the staff…. I love my Wednesday afternoons. (P4)

You can feel a lot more competent in what you're doing [when] you're working in your specialty. (P7)

Residents enjoyed developing meaningful relationships with their patients and coworkers during family medicine rotations. Rotations with clear expectations and feedback were felt to protect against burnout, particularly specialty rotations with objectives tailored to family medicine. Participants also emphasized the importance of supportive faculty and programs.

Knowing that I could [speak with faculty] if I wanted to, I think is very protective, and to know that they do hear what you're trying to say… I feel like there's a cocoon within the <site> program where the faculty are so supportive of you, and they constantly remind you of that, and I think that's part of why I like it. (P9)

A supportive family was identified as protective against burnout. Family members helped residents debrief their day and engage in activities unrelated to medicine.

### Suggestions for decreasing rates of resident burnout

Most suggestions corresponded with mitigating the contributing factors or bolstering the protective factors described above. Increasing the length of training for family medicine residency was suggested by both international medical graduates.

I think that the two years are not enough for family practice, [to meet] the current expectations, the current level of knowledge that you’re supposed to have and the current level of skills. (P3)

Other participants felt that burnout could be lessened by a medical culture that placed higher value on work-life balance and self-care. This included the need to address excessive work hours and allowing residents increased control over their schedules. One resident suggested offering ‘flex days’ which could be used at the first sign of burnout.

Instead of waiting until you’re sick, because you’ve been so decompensated for so long, because you’ve run yourself ragged and then you get sick and you have to call in sick … it’d be nice just to have a pre-emptive mental health day once in a while to just regain your composure. (P1)

Residents also requested additional support services throughout training, including access to counselors, psychologists or group sessions.

If we could all have a personal counselor... or something in that nature... a venue for us to come together and support each other, learning from our mistakes or moving forward so that we don't have to sit with that for the rest of our lives. (P7)

Program support and training for physician mentors was also suggested along with establishing an ongoing process for eliciting resident feedback and implementing changes.

More communication, more reform and value placed on the feedback that the program gets about residency experience - and demonstrating a willingness to address those concerns. (P10)

## Discussion

This qualitative study on resident burnout contributes to the existing, mostly quantitative, literature by offering a rich, in-depth description of the experience of burnout in family medicine residency and identifying contributory and protective factors, as described by residents. All participating residents described experiencing burnout at some point during their training. Similar to the participants in the Satterfield *et. al.* study, our participants expressed feelings of guilt, anxiety and frustration.^21^ Our results also support the literature reporting lack of control over scheduling, lack of time for leisure, family and friends and time demands as contributing to burnout.^3^,^16^,^19^,^20^ This study further elaborates on these factors and identifies the role of heavy workload in addition to long work hours, and residents feeling isolated from, as well as insufficient time for, family and friends as contributing to burnout. Unlike other studies, neither financial concerns were cited as contributing to burnout nor was increased financial support suggested as a means to reduce burnout.

Our results indicate that burnout is a complex and multifactorial phenomenon. Our participants identified and emphasized more systemic and program level factors as contributing to burnout than individual level ones. This is consistent with Maslach and Leiter’s work on developing interventions to combat burnout, which emphasizes the importance of the worker-workplace interface and of situational and organizational interventions in addition to individual ones^1^,^26^. Some of the contributory factors identified, such as an unsupportive medical culture and heavy workloads, will require collaboration between many agencies to resolve. Others however, such as frequent rotation changes and responsiveness to resident feedback, could be addressed by individual residency programs. Notably, protective factors elicited did not include the current resident wellness curriculum. Although training sites have different curricula, all offer between one and three lectures focused on self-care or resident wellness and provide residents with a list of resources for resident resilience, including the BC Physician Health Program and 24 Hour Suicide Crisis Line. Residents’ suggestions for decreasing burnout rates further emphasized the importance of system and program level changes. These include allowing residents more control over their schedules, offering access to counseling services, support for training physician mentors, work hour limits and a medical culture that places higher value on work-life balance.

Our study identifies a number of aspects of burnout that may be unique to family medicine training, and provides some insight into the high burnout rates found in UBC family medicine residents. Key risk factors for burnout considered specific to family medicine training were: 1) frequent off-service training, 2) devaluing of family medicine in the medical culture, and 3) discrepancy between goals of learning and actual learning on non-family medicine rotations. Compared to other specialty training programs, family medicine residents spend more of their first year on off-service rotations. During these rotations, they report hearing demoralizing and negative comments towards family medicine and experiencing a mismatch between their learning objectives and the learning opportunities available. Tailoring the structure of off-service rotations to meet the future practice needs of family physicians is likely to improve residents’ sense of personal accomplishment. Shifting the image of family physicians towards valued members of the medical team is also important.

In contrast to what they experienced off-service, participants in this study found their family medicine rotations helped them build confidence. They noted the protective nature of the longitudinal family medicine clinic, where they developed ongoing relationships with staff, preceptors and patients. These results are consistent with research showing that the development of professional confidence is protective against burnout.^21^ The importance of continuity of care in family medicine was also highlighted in a recent qualitative study of family physicians and residents. Participants indicated that continuity of care and the establishment of long-term relationships with patients enhanced family physicians’ feelings of professional competence and fostered personal growth.^27^ The inability to form long-term patient relationships, a core value of family medicine, could help to explain the distress residents experience during frequent rotation changes and constantly shifting patient lists while on off-service rotations.

This study has some important limitations. Participant recruitment and focus group scheduling posed a challenge; only ten residents were able to attend the focus group, despite more expressing interest in participating. It is thus possible that thematic saturation was not achieved. For similar reasons, although attempts were made to maximize the diversity of our sample, the results likely over represent an urban training experience and underrepresent male residents. However, the relative underrepresentation of male residents in our sample must be viewed in the larger context of a lower representation of men in family practice residency training (e.g. men made up only 25% of residents at the two UBC urban program sites in 2012). Finally, additional research is needed to confirm if these findings persist in family medicine training programs that are more predominantly rural, and those in other provinces.

Our results support a growing interest in advocating for improving medical residents’ work-life balance, both as a means to reduce burnout and to improve patient safety.^28^ To achieve this, more research is also needed to determine how best to implement the suggestions elicited by this study and whether targeted interventions at the program level will translate into lower rates of burnout. Further insight could be gained with a follow-up study after residents’ first few years in practice to see how the experience of burnout changes following transition to practice and whether burnout in residency predicts physician burnout in future. Unfortunately, burnout may not be limited to residency; Lee *et. al* show that practicing family physicians in Canada also experience high rates of burnout.^29^

### Conclusion

Resident burnout poses a significant problem: it affects over two thirds of family medicine residents in BC and has wide ranging consequences for patient care, the well-being of residents and their educational experience. This study highlights a number of potentially unique factors relating to burnout in family medicine residents. Program directors, rotation directors and all physicians with teaching responsibilities should be aware of resident burnout and take steps to implement the suggestions outlined above. While systemic and program level changes may be more difficult to implement than individual level interventions, they may also have more potential to decrease rates of resident burnout.

## Figures and Tables

**Table 1:** Participants’ demographic information

		*N = 10*
Gender	Female	9 (90%)
	Male	1 (10%)

Age	25–29	6 (60%)
	30–34	3 (30%)
	35–39	1 (10%)

Year of residency	PGY 1	4 (40%)
	PGY 2	6 (60%)

Marital Status	Single	5 (50%)
	Common-law	2 (20%)
	Married	3 (30%)

Children	No	7 (70%)
	Yes	3 (30%)

Student Loans	< $50,000	6 (60%)
	$50,000 – $150,000	1 (10%)
	>$150,000	3 (30%)

Residency site	IMG program	2 (20%)
	St. Paul’s Hospital	5 (50%)
	Vancouver Fraser	1 (10%)
	Rural sites (two distinct)	2 (20%)

PGY = Postgraduate Year

**Table 2:** Maslach Burnout Inventory scores for residents who met burnout criteria

	Residents meeting burnout criteria (2012 focus groups) *(n = 7)*	

Burnout Domain	Mean score	Category (qualifying scores)^25^

Professional Efficacy	27.0	Moderate (24 – 29)
Emotional Exhaustion	18.4	High ( ≥ 16)
Depersonalization/Cynicism	14.1	High (≥ 13)

**Table 3:** Overall resident scores on the Maslach Burnout Inventory

	2012 Focus Groups *(n = 10)*		2011 UBC Survey^23^ *(n=109)*	

Burnout Domain	Mean score	Category^25^	Mean score	Category

Professional Efficacy	27.1	moderate (24–29)	29.5	moderate (24–29)
Emotional Exhaustion	16.3	high (≥ 16)	19.3	high (≥16)
Depersonalization/Cynicism	13.9	high (≥ 13)	8.16	moderate (6–12)

Prevalence of burnout*	**70%**		**74%**	

* Defined as high risk group for Emotional Exhaustion or Cynicism

## References

[1] Maslach C, Schaufeli W, Leiter M, Fiske S, Schacter D, Zahn-Waxler C (2001). Job burnout. Annu Rev Psychol.

[2] Rafferty JP, Lemkau JP, Purdy RR, Rudisill JR (1986). Validity of the Maslach Burnout Inventory for family practice physicians. J Clin Psychol.

[3] Prins JT, Gazendam-Donofrio SM, Tubben BJ, van der Heijden FMMA, van de Wiel HBM, Hoekstra-Weebers JEHM (2007). Burnout in medical residents: a review. Med Educ.

[4] Martini S, Arfken CL, Churchill A, Balon R (2004). Burnout comparison among residents in different medical specialties. Acad Psychiatry.

[5] Shanafelt TD, Bradley KA, Wipf JE, Back AL (2002). Burnout and self-reported patient care in an internal medicine residency program. Ann Intern Med.

[6] Lindblom KM, Linton SJ, Fedeli C, Bryngelsson I-L (2006). Burnout in the working population: relations to psychosocial work factors. Int J Behav Med.

[7] Prins JT, Hoekstra-Weebers J, Van de Wiel H, Gazendam-Donofrio SM, Sprangers F, Jaspers FC (2007). Burnout among Dutch medical residents. Int J Behav Med.

[8] Thomas NK (2004). Resident burnout. JAMA.

[9] van der Heijden F, Dillingh G, Bakker A, Prins J (2008). Suicidal Thoughts Among Medical Residents with Burnout. Arch Suicide Res.

[10] Rosen I, Gimotty P, Shea J, Bellini L (2006). Evolution of sleep quantity, sleep deprivation, mood disturbances, empathy, and burnout among interns. Acad Med.

[11] Abdulaziz S, Baharoon S (2009). Medical residents’ burnout and its impact on quality of care. Clin Teach.

[12] Legassie J, Zibrowski EM, Goldszmidt MA (2008). Measuring Resident Well-Being: Impostorism and Burnout Syndrome in Residency. J Gen Intern Med.

[13] Ashkar K, Romani M, Musharrafieh U, Chaaya M (2010). Prevalence of burnout syndrome among medical residents: experience of a developing country. Postgrad Med J.

[14] Fahrenkopf AM, Sectish TC, Barger LK, Sharek PJ, Lewin D, Chiang VW (2008). Rates of medication errors among depressed and burnt out residents: prospective cohort study. BMJ.

[15] West CP, Tan AD, Habermann TM, Sloan JA, Shanafelt TD (2009). Association of resident fatigue and distress with perceived medical errors. JAMA.

[16] Ishak WW, Lederer S, Mandili C, Nikravesh R, Seligman L, Vasa M (2009). Burnout During Residency Training: A Literature Review. J Grad Med Educ.

[17] McNeeley MF, Perez FA, Chew FS (2013). The Emotional Wellness of Radiology Trainees. Acad Radiol.

[18] Eckleberry-Hunt J, Lick D, Boura J, Hunt R, Balasubramaniam M, Mulhem E (2009). An exploratory study of resident burnout and wellness. Acad Med.

[19] Lemkau JP, Purdy RR, Rafferty JP, Rudisill JR (1988). Correlates of burnout among family practice residents. J Med Educ.

[20] Galam E, Komly V, Le Tourneur A, Jund J (2013). Burnout among French GPs in training: a cross-sectional study. Br J Gen Pract.

[21] Satterfield JM, Becerra C (2010). Developmental challenges, stressors and coping strategies in medical residents: a qualitative analysis of support groups. Med Educ.

[22] Purdy RR, Lemkau JP, Rafferty JP, Rudisill JR (1987). Resident physicians in family practice: who’s burned out and who knows?. Fam Med.

[23] Laramee J, Kuhl D Burnout and Suicide Prevention among UBC Family Practice Residents.

[24] Kitzinger J (1995). Qualitative research. Introducing focus groups. BMJ.

[25] Maslach C, Jackson SE, Leiter MP, Schaufeli W, Schwab RL Maslach Burnout Inventory Instruments and Scoring Guides. Forms: General, Human Services & Educators.

[26] Maslach C (2003). Job Burnout: New Directions in Research and Intervention. Current directions in psychological science.

[27] Schultz K, Delva D, Kerr J (2012). Emotional effects of continuity of care on family physicians and the therapeutic relationship. Can Fam Physician.

[28] Edwards S (2011). Resident Wellness and Work/Life Balance in Postgraduate Medical Education.

[29] Lee F, Stewart M (2008). Stress, burnout, and strategies for reducing them. Can Fam Physician.

